# Using computer decision support systems in NHS emergency and urgent care: ethnographic study using normalisation process theory

**DOI:** 10.1186/1472-6963-13-111

**Published:** 2013-03-23

**Authors:** Catherine Pope, Susan Halford, Joanne Turnbull, Jane Prichard, Melania Calestani, Carl May

**Affiliations:** 1 Faculty of Health Sciences, University of Southampton, Southampton SO17 1BJ, UK; 2 Faculty of Social and Human Sciences, University of Southampton, Southampton, SO17 1BJ, UK; 3 Faculty of Medicine, University of Southampton, Southampton, SO17 1BJ, UK

**Keywords:** Computer technology, CDSS, Urgent care, Emergency care, Normalisation process theory

## Abstract

**Background:**

Information and communication technologies (ICTs) are often proposed as ‘technological fixes’ for problems facing healthcare. They promise to deliver services more quickly and cheaply. Yet research on the implementation of ICTs reveals a litany of delays, compromises and failures. Case studies have established that these technologies are difficult to embed in everyday healthcare.

**Methods:**

We undertook an ethnographic comparative analysis of a single computer decision support system in three different settings to understand the implementation and everyday use of this technology which is designed to deal with calls to emergency and urgent care services. We examined the deployment of this technology in an established 999 ambulance call-handling service, a new single point of access for urgent care and an established general practice out-of-hours service. We used Normalization Process Theory as a framework to enable systematic cross-case analysis.

**Results:**

Our data comprise nearly 500 hours of observation, interviews with 64 call-handlers, and stakeholders and documents about the technology and settings. The technology has been implemented and is used distinctively in each setting reflecting important differences between work and contexts. Using Normalisation Process Theory we show how the work (*collective action*) of implementing the system and maintaining its routine use was enabled by a range of actors who established *coherence* for the technology, secured buy-in (*cognitive participation*) and engaged in on-going appraisal and adjustment (*reflexive monitoring*).

**Conclusions:**

Huge effort was expended and continues to be required to implement and keep this technology in use. This innovation must be understood both as a computer technology *and* as a set of practices related to that technology, kept in place by a network of actors in particular contexts. While technologies can be ‘made to work’ in different settings, successful implementation has been achieved, and will only be maintained, through the efforts of those involved in the specific settings and if the wider context continues to support the coherence, cognitive participation, and reflective monitoring processes that surround this collective action. Implementation is more than simply putting technologies in place – it requires new resources and considerable effort, perhaps on an on-going basis.

## Background

Governments and corporations – both private and public – have placed great hope and expectation in the capacity of technological innovation in general, and information and communication technologies (ICTs) in particular, to play a significant role in overcoming the structural tension of rising costs and the need for fiscal restraint in the delivery of public healthcare services [1-4]. Digital ICTs have been enrolled in telemedicine to enable remote consultations [5,6] and telemonitoring to allow patients to undertake routine testing and data transmission and enabling remote surveillance by clinicians or clerical workers [7,8]. For all this enthusiasm, the significant gap between the high hopes and expectations of policy-makers and managers and the actual practice of implementation of these technologies has been highlighted by research which has consistently revealed an extensive catalogue of delays, compromise and failure [9-14]. It appears that technological interventions do not slip seamlessly into established practice but instead they meet with resistance and rejection because they threaten established work and organisational routines or clash with established professional identities and power relations [15-20] We also know that interventions that ‘work’ in one setting or speciality may not work elsewhere [21,22].

Much research about health technologies has sought to understand why interventions do, or more frequently do not, get embedded in practice [10,23,24]. A great deal of research in this area has been characterised by an assumption that there is an ‘it’ (i.e. a technology) that is implemented – rather than seeing the ensemble of work practices and social relations that shape each other to bring a technology into use. This focus has helped to identify a variety of barriers and facilitators to successful implementation but less attention has been paid to wider strategic, political or workforce issues. In terms of research design, with a few notable exceptions [25,26] this work has relied on case studies focussing on specific occupational groups (e.g. doctors or nurses) and their response to particular technological interventions. While this has been excellent at revealing the complex and particular processes that shape particular outcomes, it has not enabled systematic analyses of wider issues, especially the tricky problems associated with transferring a technology from one setting to another.

So, while policy pushes the case for new technologies to deliver healthcare improvement, the empirical literature continues to highlight an implementation gap. We ‘know’ that there are myriad barriers to success. This paper reports a project which explored how an innovative ICT was successfully brought into use by a range of different staff in different settings. Specifically we try to understand how a particular computer technology becomes normalised; how a technology becomes embedded in work practices and in different healthcare settings.

### Computer decision support systems in healthcare

The focus of our study was a single technology, a computer decision support system (CDSS) deployed in different emergency and urgent healthcare settings. CDSS are computer programmes designed to assist with decision-making. CDSS typically combine an expert knowledge base with an algorithmic or inference based set of rules which guide or inform decisions. CDSS have become increasingly popular in healthcare, and have diffused rapidly across the NHS, for example to support GP prescribing e.g. ‘PRODIGY’, [27] and diagnosis and treatment decisions by doctors and nurses e.g. Odyssey ‘TAS’, [28]. In clinical consultations these technologies are used to structure questioning, gather patient information and to synthesise these with clinical knowledge to support or inform diagnosis and treatment decisions. CDSS have been developed for use by non-clinical staff, notably in emergency medical care where they have been successfully used to help manage and prioritise the dispatch of ambulances. CDSS are promoted as helping to standardise and structure work, allowing staff to work more efficiently, safely, or faster, or enabling substitution or reallocation of tasks [29].

The CDSS of interest here is used to support the provision of a telephone-based service in which call-handlers assess and prioritise calls from patients (or their carers) to assign them to appropriate care provision. This CDSS is an expert system built on an extensive clinical evidence base subject to a continuous process of evidence review and update. A series of logical algorithms (pathways) underpin questions which a call-handler uses to ask the caller/patient to determine the clinical skills required to provide care, and the timeframe in which they must be accessed. The call-handlers work in a large open plan office (call centre) and sit at a desk facing two computer screens. Each has a headset to receive incoming telephone calls and works through questions on the screens, clicking on appropriate ‘answers’ and typing free text information as necessary. The particular CDSS studied offers some flexibility in how the questions are phrased, and prompts are suggested which allow the call-handlers to probe for details and work through the linear algorithm or ‘pathway’ and reach a ‘disposition’ - a recommended course of action, which can include immediate 999 ambulance request, referral to a range of different primary care services, or suggestions for self-care. Call-handlers typically work 10–12 hour shifts and cover the whole 24 hour period. In addition to the call handlers a number of other staff work in or near the call centre area (supervisors, clinical support staff and trainers). Each of the call centres in this study was located with or in a larger healthcare organisation (NHS Ambulance Trust or Primary Care service) and this meant that that senior managers and other health service professionals and staff worked nearby.

The literature on this type of healthcare call-handling work is necessarily limited as this is a relatively new mode of healthcare provision. To date published work in this field has mainly looked at nurse-led call-handling [30,31] in part because the extension of this telephone-based service to a non-clinical workforce is so new. To our knowledge, the research reported here is the first comparative case study of a CDSS used by clinical *and* non-clinical call-handlers in different healthcare settings.

Of course, computer assisted call-handling has a longer history in settings outside healthcare, notably the finance and insurance sectors. Contemporary sociological critiques of this type of work have pressed the case for seeing call-handling in the context of globalisation of capitalism and labour substitution [32], Taylorised mass production and control [33] and in terms of power and resistance [34]. While these analyses are powerful, and have highlighted aspects of expertise, performativity and normative constraint, we nonetheless felt that it was important to explore how digital ICTs were being enrolled in the everyday practice of healthcare work. In particular we sought an approach that would focus on the collective social action of call-handling and technology use and show how workforce relations were located within the ‘special’ context of health services and the particular social norms that frame and constrain this sphere of action.

### Research approach

The CDSS we studied was designed to allow non-clinical staff (as well as clinical staff), to make comprehensive clinical assessments of telephone callers to emergency and urgent care services. We carried out a cross-case comparative study this new technology in three different healthcare settings: ‘999’ emergency care, a GP out of hours (‘OOH’) urgent care service, and a new ‘single point of access’ (‘SPA’) service for urgent and unscheduled care.

We drew on Normalization Process Theory, or NPT [35,36] to provide a theoretical lens for our empirical and methodological analyses. NPT is concerned with ‘how and why things become, or don’t become, routine and normal components of everyday work’ (36 p 535) and it defines four core mechanisms that shape the social processes of implementation, embedding and integration of ensembles of social practices. These dynamic and interrelated domains are ‘*coherence’* – the extent to which an intervention is understood as meaningful, achievable and desirable; ‘*cognitive participation’* – the enrolment of those actors necessary to deliver the intervention; *collective action* – the work that brings the intervention into use; and ‘*reflexive monitoring’* – the on-going process of adjusting the intervention to keep it in place. We used these domains to provide a framework for our findings in each of the three case studies and as a way of integrating our findings across the three cases.

The choice of NPT was a deliberate one. Our research team included social scientists from sociological, psychological and health services research backgrounds and we sought a robust theoretical framework for our analysis which could be practically and appropriately applied to the comparative study of the CDSS in the chosen settings. We were familiar with a number of theoretical approaches which might be loosely collected under the heading ‘Science and Technology Studies’ (STS) which had grown out of sociological research and parallel Sociotechnical network approaches which drew on social informatics and computer science. Both of these perspectives attempted to go beyond a naive technological determinism and argued instead that technologies such as ICTs should be understood as socially shaped and comprised of dynamic interrelationships between people, artefacts, practices and structures. Reviewing these fields we considered using Actor Network Theory [37,38] but discounted this because we felt that while this helpfully alerted us to the importance of networks of human and non-human actors it focussed more on the performative aspects of technology in use and had less to say about the normative constraints and the environment (with perhaps the notable exceptions of Callon’s [39] study of markets and MacKenzie’s [40] classic examination of the nuclear missile industry). Similarly while sociotechnical interaction networks [41] helpfully capture the social co-construction of technologies and wider ecology of the networks surrounding them we were minded to concur with Meyer’s [42] assertion that these provided a research strategy rather than theory.

We also considered stances that apply psychology and economics to the problem of understanding technologies in use. These include the Theory of Planned Behaviour [43] and the Theory of Rational Action/Rational Choice Theory [44] both of which assure the centrality of action. We were concerned that these tended to focus on human behaviour (and neglect non-human actants which we felt were important) and over-emphasise instrumentality. While these approaches had a considerable track record in experimental research they appeared to perform less well when applied to ethnographic research of the type we were proposing. Likewise the Technology Acceptance Model [45] appears to offer a hybrid of rational action approach and diffusion theories [46,47] which can explain adoption, appears less well suited to examining embedding and normalisation.

NPT provided a middle range theory designed explicitly for examining the types of phenomena we were interested in, namely technologies in everyday use, the work of normalisation and the practices of embedding [48]. As the theory is relatively new, we were also keen to explore the use of the constructs of this theory as a structured approach to data interpretation to add to the early corpus of empirical applications of this approach to demonstrate how it might align with the other theoretical perspectives we have noted above.

## Methods

Ethical approval for this study was given by the Wiltshire Research Ethics Committee (REC 08/H0104/56) in August 2008.

This paper focuses on ethnographic data from our study. This method was chosen to capture the complexities of social interaction in a naturalistic rather than experimental way. Greenhalgh and Swinglehurst [49] provide a particularly helpful discussion of the merits of this approach for studying ICTs and organisational practices. We used non-participant observation conducted over 20 months, at different times and days of the week across the three sites. This observation focused on the call-handlers using the CDSS but we also observed other staff, including clinical supervisors (999 and SPA site), ambulance dispatchers (999), GPs (OOH) and their interactions with each other and the CDSS. The observation was purposively structured to capture activity at different times of day/days of the week and covered all or part of a shift depending on the setting (between 4 and 8 hours). The observational fieldwork included informal conversations with staff and in addition we interviewed staff and stakeholders (policy makers, commissioners, system developers, managers) using a semi-structured topic guide developed from our knowledge of the relevant literature and initial observations. Detailed notes of each observation period were overtly taken and transcribed soon afterwards and these included verbatim or near verbatim statements. All details about the nature of calls and the triage process were anonymised.

Call-handler interviewees were purposively sampled and interviews were conducted during working hours at points in the day/evening when the service was less busy (with the agreement of shift managers). They were given the study participant information leaflet and time to consider if they would like to participate. Stakeholders and managers (including policy-makers, commissioners, system developers, corporate and operational managers) were also approached and provided with a participant information sheet and a time for the interview was arranged if they agreed. Call-handler interviews typically took 30–45 minutes because of the constraints of their work patterns, other interviews were between 60–90 minutes. Where possible, interviews took place in a private office or meeting room but in OOH some interviews took place in the call centre room. All the interviews were recorded. The interview topic guide was adjusted to reflect the experience and seniority of each participant. Alongside the observation and interviews we collected documents concerning the CDSS design, training materials and reports and this was used to inform our analysis.

### Analysis

The data were analysed, initially via systematic reading and open coding, undertaken independently by five members of the research team and then in collaborative meetings [50] which allowed discussion of emerging themes. A sixth member of our team (CM) had been closely involved in the development of NPT so as a way of enhancing the validity of the coding we deliberately excluded him from this analytical process to reduce the possibility that we would simply ‘fit’ the data into the domains offered by NPT. Our coding discussions formed the basis of an agreed coding frame, and where there were disagreements about codes we attempted to resolve these amongst the coding team calling on CM for advice and clarification about the theory to inform this consensus as necessary (whist there were a number of early disagreements in the coding process we found that were able to resolve these satisfactorily amongst the team). We used NPT as a framework for the coding and analysis combined with a thematic approach, using constant comparison [51] to examine codes and refine emerging themes to augment the understanding offered by the NPT domains. To support the analysis we wrote narrative data summaries and used matrix/charting [52,53] techniques to facilitate comparison. As the study progressed, the analysis was structured to examine all the data within each setting, and then across the settings using our research questions and the framework provided by Normalization Process Theory.

## Results and discussion

We collected nearly 500 hours of observational data over approximately 35 days at each site between November 2008 and August 2010 (Table 1). A total of 61 interviews were conducted, with a total of 64 respondents (Table 2). Below we present the analysis of these data for each site in turn before discussing the implications of these findings for understanding the implementation and embedding of this technology.

**Table 1:** Observational data - time spent in each setting

**Setting**	**Number of hours**	**Number of days**	**Time period**
999	170	41	Nov 2008 – Jul 2010
SPA	172	33	Oct 2009 – July 2010
OOH	149	27	Sept 2009 – Aug 2010
Total	491	101	Nov 2008 – Aug 2010

**Table 2:** Interviewees by role and location

**Staff /stakeholder group**	**Location**				
	**999**	**SPA**	**OOH**	**External**	**all**
Call handler	6	15	12	1	34
Call supervisor/ manager	3	4	3	1	11
Other call centre staff	1	n/a	n/a	1	2
Clinical	3		1		4
Senior manager	6		1	1	8
CDSS Developers	n/a			2	2
Government level	n/a			3	3
Total	38		17	9	64

### Emergency call-handing for ‘999’

Prior to the introduction of the CDSS in the ambulance service, 999 emergency calls were handled by non-clinical staff using a basic classificatory system (Criteria Based Dispatch) which meant taking a name and address and, usually, dispatching an ambulance. In contrast, the CDSS offered a comprehensive triaging system built on a clinical evidence base, for use –by call-handlers who receive 12 days training on using the computer system and are not required to have clinical training. The CDSS was promoted by the Department of Health, who had trialled it and vouched for its safety but it was also supported by ambulance service managers, and call-handlers themselves, keen to dispatch ambulances more appropriately:

The benefits for me were not taking some people to hospital and only responding vehicles to people who really needed it. (Control room manager)

It wasn’t that long ago a good ambulance service took everyone to hospital but now it’s all changed. Now it’s about how many patients we can keep out of hospital safely. (Senior Manager)

These beliefs were tied to imperatives for rationing, in the face of rising demand and seen as an opportunity to discipline patients, especially ‘inappropriate’ callers with non-emergency problems, and also to support for ‘evidence based medicine’ – whereby formal knowledge bases (systematic reviews, expert systems, etc.) based on the accumulation of good practice replaces knowledge vested in and accumulated by individual clinicians. Trust in the system was high, because it was built on this externalised expert knowledge, making it safe for non-clinicians to use:

Call-handler explains that the CDSS has been designed by ‘a huge group of people…non-clinicians, clinicians - lots of input…it’s a very safe system to use, used correctly’. (Observation notes)

Drawing on NPT we can say that the combined rhetorics of safety, rationing and medical expertise meant that the *coherence* of the innovation was strong: it made sense for the range of participants involved, from policy makers to individual call-handlers. However, coherence alone does not explain why the technology was successfully brought into everyday use: in principle support for innovation has to be translated into participation and action. Interestingly, despite the general communal specification of coherence, each of the actors participating in the implementation of the CDSS did so for distinctive reasons. For the Department of Health, the CDSS offered the chance of a high profile technological success – much needed given all the promises about harnessing technology to achieve NHS modernisation. This general goal was shared by the CDSS developers, but they were keen – of course – to see their particular CDSS used, and were prepared to work very hard to make it work in this site. The Ambulance Trust managers also had specific local ambitions:

We’ve always been in the forefront of new things, new initiatives … projects. …you can guarantee if something’s going to happen, we will be at the forefront of that and we’ve got a reputation for that, of wanting to try new things and wanting to push the boundaries a little bit further, so, it didn’t surprise us when we won the pilot for [the CDSS]. (Senior Manager)

For the call-handlers, there was less choice about participation – their use of the CDSS was imposed on them by their managers. However, we know from previous studies that this is not always sufficient to make an intervention work e.g. [54]. The active buy-in of the call-handlers was important to successful implementation. In practice, this was achieved because of a strong commitment amongst the call-handlers to disciplining ‘time-wasters’ (the inebriated and those with minor ailments who should not be calling 999), although alongside this the call-handlers had some concerns that the CDSS might ‘de-skill’ them (something that we return to shortly). In addition to the human actors enrolled at 999 the CDSS had to link to other technological devices systems and geographical and demographic databases – for example maps showing incident and ambulance locations - in order to ‘perform’ the work of 999 call-handling. To summarise in NPT terms, we can see how *cognitive participation* – the enrolment of the actors necessary to make the intervention work – took place in this case. Notably, attention to cognitive participation shows that diverse motivations for participation were contained within the broader discursive formation of coherence.

So far, so good, strong coherence and cognitive participation were established around the intervention. However, bringing the CDSS into use required concerted action from all the actors involved, particularly the call-handlers who had to use the system in their everyday practice. Here we found that whilst the system was effectively brought into use, it was not in the way envisaged at the start: making it work involved some surprising compromises. Specifically, whilst the CDSS ‘promised’ a combination of Evidence Based Medicine and de-skilling, in practice the call-handlers’ experienced up-skilling – learning to navigate multiple information systems, and engaging in extended and often very difficult conversations with callers:

[The CDSS] is harder, and it’s easier, if you know what I mean. It’s harder because you’re having to get a lot more information and you seem to, sort of, everything’s on top of you … (Duty Manager)

Furthermore, while the CDSS was expected to control the knowledge in play the call-handlers continued to draw on and develop experiential knowledge and exercise discretion around personal and organisational values not programmed into the software. In the following example, a call-handler over-rode the CDSS disposition, to allow an ambulance dispatch where none was indicated. She says:

… “that was naughty…sometimes you let your heart rule your head”. […] There were two main reasons why she overrode the disposition 1) she was an elderly lady who was anxious, 2) there were difficulties in communicating - both her speech and her hearing - was making it a lengthy procedure. The patient had already spoken to [another service] and to the ambulance service. If the call was referred on the patient would be required to speak to a third agency. She felt that this call was going to take a long time to resolve, potentially leading to further distress for the patient. She [the call-handler] justified her actions by saying “I haven’t done an override recently…” (Observation notes)

The development of experiential expertise and the use of tacit knowledge and discretion has of course been highlighted in other organisational research such as the call centre studies noted earlier, and in classic studies like Suchman’s analysis [55] of how creative improvisational reasoning underpins ‘plans and situated actions’ . What is novel here is that this CDSS offered these *non-clinical* staff an opportunity to perform *clinical* identity and gain some additional status and authority. Despite the introduction of an extensive auditing system, these discretionary practices and outcomes were tolerated. In NPT terms, we see the importance of the wider institutional setting within which the collective action is taking place: how implementation takes place at the point of interaction between established and emergent rules, practices and identities. The call-handlers effectively operationalized the innovation and built shared understandings of how this could be done, but this was not exactly as expected. Indeed, tolerance for unanticipated outcomes and willingness to moderate expectations and processes was key to embedding the CDSS in this site.

Throughout the implementation process, the Department of Health, the developers and the Ambulance Trust were attentive to the need for incremental adjustments, a process referred to as reflexive monitoring within NPT. For example, whilst extensive call-audit procedures were introduced alongside the CDSS, which could have been used to ‘police’ the call-handlers very closely, in practice these procedures operated with a tolerance that allowed call-handlers to adapt phrasing and use some of their ‘own’ knowledge in managing calls. This call-handler discretion helped make the system work and possibly also reduced staff turnover by making the job more satisfying. The managers also found it necessary to introduce new ‘clinical supervisors’ (typically a nurse or paramedic) to support the call-handlers in dealing with complex or non-standard cases. Whilst the developers tried to accommodate these kinds of cases within the software, this proved ineffective, and the presence and actions of clinical supervisors effectively legitimised ‘fuzzy logic’ in the day-to-day operation of the CDSS. The Trust also provided additional clinical training for the call-handlers to help them understand symptoms (e.g. explaining what happened when someone had a stroke). This, combined with the presence of clinicians reinforced the sense that call-handling work has a strong clinical component, making their work both possible and rewarding. The interactions between these human actors and the technology and interpretive flexibility in everyday use of the CDSS led to continual evolution of the system. We know from Science and Technology Studies [37,38] that technologies are never fixed or finished and this was true at 999 where the CDSS developers continued, some 2–3 years into deployment, to upgrade and adapt the system.

### Single point of access to urgent and unscheduled care

Single Point of Access (SPA) was a new service, using the CDSS to triage callers and direct them to the relevant primary care services. The call-handler was able to book appointments with appropriate care professionals (commonly a GP) and could also book patient transport for this. The call-handler could also offer self-care advice, prescribed within the CDSS, if indicated. The service was operated by the same ambulance trust as 999 above, as a new venture – both for them (this was the first time they had delivered non-emergency services) and more generally (this was the first attempt to link primary care services in this way).

As for 999, the CDSS was seen as a way to improve healthcare delivery, specifically as a way to integrate and rationalise out-of-hours services. This was an important Department of Health priority that resonated with local managers:

…we’d already realised that, … the whole health care model’s becoming so complicated it’s difficult to navigate your way, way through it; it’s not the patient’s fault that they don’t know how to, how to access and who to access and that the NHS… should get its act together and make this as easy as possible. (Senior manager)

Based on their experience with 999 the managers were confident in the CDSS and, indeed, believed that they could employ staff with the same skill set as 999, and that this would – eventually - enable staff to work flexibly across urgent and emergency care, maximising effective workforce management.

I would like to think that they would all …be skilled to take all levels of calls. I think if we start to carve out different types of calls coming to different, then you sort of lose that, the capacity. If they are all trained to take the 999 calls, then when they’re not doing an urgent call, they can pick up a 999 call and vice versa. So I think it’s, you know, you have better use of resources if they’re all trained up to take all calls. (Senior manager)

For the developers SPA was an opportunity to extend the use of their system, to demonstrate its value more widely. For senior managers, the advantages of the CDSS were even greater than in emergency care:

The big prize will be to apply it to those callers who would previously have phoned their GP in-hours because they had an unscheduled care need or they would have phoned their doctor out of hours…or they would have phoned NHS Direct… And my belief is that [the CDSS’s] true worth will then be… truly exhibited. (Senior manager)

Again, framing this using the domains of NPT, the intervention made sense to the key actors in SPA because of commitment to integrated care. But the need for coherence and buy-in was rather different in this case, compared with 999. First, because successful implementation required buy in from primary care and community services and, as part of this, integration of the CDSS with a range of external technological systems (e.g. appointment booking). And second, because the service recruited new call-handlers, who – by default – did not share any of the 999 call-handlers’ reservations about the CDSS and had no pre-existing practices to be adjusted or accommodated.

Nonetheless, the everyday practice of SPA call-handling using the CDSS proved to be highly complicated as different pathways through the CDSS algorithms resulted in different dispositions (e.g. call an ambulance, wait and see your GP) so that achieving an agreed outcome with the caller could be difficult:

… the final disposition was a 19 minute ambulance. The call-handler explained that an ambulance was going to be on its way but the patient did not want an ambulance: she refused it. So the call-handler called the clinical supervisor and clicked on early exit. He then arranged an appointment at one of the primary care centres at 6.50pm, by selecting a PCC from the list and clicking on the option book appointment. He confirmed the appointment to the caller and then filled in a box on care details with some brief comments on the case. (Observation notes)

A conversation between a developer, clinical supervisor and researcher comparing 999 and SPA. The clinical supervisor mentions that she thinks that the work requires a “different skill set”. The developer thinks that, in some ways, the out-of-hours work is more difficult, and describes it as having “more complexity”. She says that some call-takers might be suited to one type of work rather than the other. She characterises the difficulties of the different types of work broadly as: on the one hand emergency work is stressful/requires thinking on your feet/acting quickly/confidently, on the other hand, out-of-hours work is complex, less straightforward, and more time consuming – requires patience. She notes that “there are some fantastic [999] call-takers” but she says that she think some of them might find out-of-hours work “a bit boring” in contrast to the “adrenaline” involved in 999 work. (Observation notes)

The everyday work that it takes to make the intervention work is different in these two different cases. In NPT terms, the *collective action* is shaped by contextual factors, including the skills required for specific forms of work, and the teamwork required to link aspects of the work (particularly important in SPA).

In the initial phase of SPA, considerable resources were allocated to ensure that problems which arose were dealt with swiftly. For example managers and developers worked shifts alongside the call-handlers during the initial implementation phase to ‘trouble shoot’ and fix problems as they arose. SPA also benefitted from the lessons learnt in 999 – for example the introduction of clinical supervisors and the handling of the audit process. In this sense, the reflexive monitoring processes, highlighted by NPT were transferrable across settings. The clinical supervisor role was also added in the SPA setting; indeed it was seen, in this context as a necessity, providing clinical input, partly because the system was viewed as necessarily over-cautious:

if you are uncertain, … you can go to a supervisor and be reassured that you are doing the correct thing. Whereas, I think, if you didn’t have a clinical supervisor you would always err on the side of caution… perhaps, sending an ambulance when you wouldn’t necessarily need one. [ …] So it's really good to have somebody who has the medical knowledge in the room, should you need them. (Call-handler)

Whilst at 999 the introduction of new practices to enable reflexive monitoring occurred alongside the introduction of the CDSS, many of the audit and monitoring practices were firmly in place when the SPA service commenced and were easily accepted by call-handlers. They reported deriving a sense of competency, of being ‘good at what they do’, from passing audits, although it is worth noting that the SPA call-handlers did not have target rates for dispositions, a particularly unpopular aspect of auditing in 999.

Thus we see that coherence about the technology had to be established at SPA, in part built on the foundational sense-making achieved in 999, but also built around new service integration and delivery. New staff had to be enrolled into bringing the CDSS into everyday use, and this entailed longer and more complex negotiations with patients, a broader range of dispositions than found at 999 and some subtly different ways of auditing and monitoring practice. Turning to our final case study, our theoretical framework allowed us to systematically examine similarities and differences once more.

### General practitioner out of hours service

Located in a different Trust, on the other side of England, the Out of Hours (OOH) service introduced the CDSS in response to new national care standards. Satisfaction with the existing system was high amongst the managers, GPs and call-handlers:

we were very happy with it; it worked very well. Like I said, we won an award … because it was so effective. The PCTs locally were very happy and they’ve audited it. And we have a clinical governance system that reviews calls and the outcomes of the calls, and the performance of the operators, the GPs, nurses, etc. (General Practitioner)

The positive, with using that system, was that the calls used to take on average 2 minutes …. with the old system, they didn’t need as many operators, so it was more cost effective. (Call-handler)

This presented a different challenge for those promoting the new system – to achieve coherence they had to persuade the relevant actors that the new system would be as good, if not better, in a context where there was little ‘shop-floor’ imperative to make a change. Consequently, the new CDSS was not presented as an alternative but as a similar way of doing the task, but in a way that met new bureaucratic requirements.

The main advantage is that it’s a nationally recognised system. It’s obviously gone through all the Royal Colleges, it’s been agreed, and so from a litigation perspective, a medico-legal perspective, it’s very safe and secure. So that gives us real security, the fact that it’s good. [ … ] But, basically, [it] is very, very similar. It works in a similar way to the way we do our [old] protocols. (Senior Manager)

In this way, the managers attempted to build on coherence around the old system to build support for the new one. This said, they were also keen to reduce demands on doctors’ time and to direct callers to alternative dispositions or offer self-care advice and this entailed additional roles for the call-handlers’ and led to some ambivalence about the CDSS:

There's nothing that I can say that I think it is better than the way we were doing it, not at the moment. It is, first line triage done by non-clinical staff, for half the price that you're going to have to pay a nurse. If that’s good, if that’s saving people money, that’s fine, but they're asking people who have 60 hours training and not a medical background at all, to do what they're doing, and sometimes I think, oh dear [laughs]. (Call-handler)

Also, unlike the other settings, the CDSS was introduced in OOH before all the call-handlers had been trained and so ran alongside the existing system for 6 months. This made buy-in more problematic – why change when there is a functioning system in place? In response the CDSS developers (themselves anxious to secure an exemplar of the CDSS in use in OOH) provided significant additional support at this site. Meanwhile, a key player from the Department of Health took it upon himself to actively promote the CDSS to key stakeholders, lending additional credibility. Nonetheless, there was resistance from call-handlers here, some of whom were concerned about how much buy-in there was amongst the range of actors implicated in delivery:

Quite often there’ll be a heated discussion going on somewhere of people really that are just running it down, and probably people that have never used it. I mean, the doctors obviously haven’t… they don’t have to use it because we’re using it, but there’s a lot of negativity about it. I don’t know what the answer is to that one, but it’s the old saying: you need to get the people in charge or the right… the people at the top need to be in, trained and confident with it before we start pushing it up at the bottom. (Call-handler)

In the everyday practice of using the CDSS, these call-handlers also had to implement extended questioning, and the time taken for each call went up. Whilst the demands were not as heavy as for the other two sites – 999 call-handers dealing with life and death emergencies and SPA call-handlers with multiple primary care services - the call-handlers in OOH still required an increased level of understanding:

You’ve got to drive it and you’ve got to have the knowledge and the background to work with that because you’ve got to understand the inference of what it is that you’re asking. If you don’t understand what you’re asking, you’re not going to be able to use it effectively. (Call-handler)

Notably, the call-handlers here had a strong commitment to one particular outcome: giving callers access a GP. This was what they had done before, and it was what they expected to do now. Whilst the CDSS was supposed to triage, and therefore deny access to some callers, the call-handlers would work ‘around’ the system, often exiting the CDSS before the final disposition:

Call-handler: If it's children’s coughs, colds and flu and things, then fine, I will […] let it go where it wants to go. And if it goes ‘to pharmacy within three days’, then that’s fine, but that’s not what the patient wants to hear. The patient has rung us up because they want to see a doctor or a nurse, not because they can see a pharmacy in three days. But I will let it take me and then they will say well no, I want to see somebody. So that’s wrong.

Q: You have an early exit and you will…

Call-handler: Yeah, give the patient an appointment. But then you know it's taking you where it's supposed to take you but the patients don’t want that, they have expectations other than seeing the pharmacist in Tesco’s in three days. (Call-handler)

Reflexive monitoring at OOH was somewhat different to 999 and SPA because existing audit systems were less developed and there were fewer staff resources. Initially there was only one member of staff responsible for all training and auditing (compared to the 999 and SPA setting, which had a team of about six trainers/auditors). Whilst all OOH call-handlers were audited in the same way as SPA and 999 they only received feedback on their performance if they failed an audit. As a consequence, some call-handlers seemed unaware whether or not they had been audited. However, despite this emphasis on using monitoring to identify bad practice, there was still acceptance of flexibility in using the CDSS. Interpretive flexibility was legitimated by general practitioners and managers, and was most apparent in call-handler decisions to not use the CDSS for certain types of calls (e.g. palliative care patients):

…for the patient who’s at end of life, [where it’s] not possible to answer questions on the telephone, and you’re asking for a specific type of pain or, and they’re obviously, they can’t answer you. It’s not appropriate to put them all the way through questioning when you wouldn’t be leaving them for two hours or six hours anyway. You would want them to be seen as quickly as possible. So there’s no point in putting them through the torment of taking a load of questions, for them to be asked all over again by the doctor. So in areas like that, it’s not really appropriate to assess them. (Manager)

As a coda to this case, towards the end of our research the OOH service took on reception work at the local Urgent Care Centre located in an acute hospital, using the CDSS to triage face-to-face encounters. Initially it was assumed that the work that would be required to implement the system in the new setting was well understood from the original OOH experience, but this was not the case. Face to face work required additional skills, including negotiating with more than one person simultaneously - often a patient came to the desk with a carer or family member, or using visual and non-verbal cues (e.g. establishing the amount of blood loss without asking the patient) to support assessment; in effect coherence and participation had to be established and reprised once again in this new environment.

### Discussion

Our analysis shows how the ‘same technology’ was used distinctively in different settings reflecting important differences in the nature of the service and care provided and the social interactions between workers, stakeholders and service users. Previous research has alerted us to the fact that the implementation of technologies depends on the interplay of technology and the workforce in everyday practice [55-57]. Our study has extended this understanding by using a theoretical framework to enable cross-case comparison and identification of the mechanisms underpinning successful implementation. We have shown, using NPT how *coherence* was achieved around the CDSS despite local context variation. Across all three sites there was agreement that the CDSS was suitable for the (varied) tasks and that appropriate resources were in place to enable effective implementation, although these varied between settings. There were differences between settings where the CDSS replaced an established system with existing staff and where the service and/or the staff were new and the work of establishing coherence had to be altered to reflect this. It was clear that knowledge, experience and work identities built through doing call-handling work influenced the coherence of the CDSS for staff in the different settings. What is especially interesting in the wider policy context – where this same CDSS is now being used to support a national ‘111’ urgent care service [58] is that coherence was not just a local ‘problem’, it was necessarily underpinned by wider understandings and discourses for example about the necessity of rationing and the need to modify caller/patient behaviour and beyond that the very legitimacy of evidence based medicine and the kinds of expert knowledge which underpinned the CDSS.

In all three settings key players were successful in enrolling a diverse network of people and technologies to bring the CDSS into use. We have shown that managers in 999 and OOH had to work harder to enrol call-handlers because of their prior use of a different technology, and the CDSS developers had higher engagement with the 999 setting which built trust and fostered enrolment and legitimation of the CDSS. Considerable effort was expended in enrolling staff although not all were in the same position regarding the CDSS – for example call-handlers had little power to resist its introduction if they wanted to retain their employment. What is important however is that the technology was implemented despite, or even because of, the emergent processes by which the call-handers and stakeholders understood, engaged with and reinterpreted implementation – a finding that is supported by a previous study of a similar healthcare decision support system [59].

Again, referring to the framework provided by NPT, we have seen how implementing and using the CDSS required collective purposive action. In 999 call-handlers used the CDSS in the management, categorisation and prioritisation of emergency calls and it was viewed positively despite the apparent *intensification* of their work. In SPA the CDSS facilitated the management of urgent care calls, sorting by urgency and enabling referral to services and/or the giving of health advice but it also *extended* this work beyond that done in 999; calls were longer and required more probing questions. In OOH the CDSS managed calls to out-of-hours care and face-to-face attendees at an Urgent Care Centre and here their work was extended and had become more *scripted.* Earlier studies of call centre operators – largely in non-healthcare settings - identified similar features and noted the demanding nature of call centre work, but this research has tended to focus on issues of management and surveillance (see for example [60,61]) rather than implementation. For the purposes of understanding implementation what is important is that deployment of this CDSS and embedding it in practice, changed the work and the workers in each setting. Call-handling used expertise based on discretion, negotiation and translation skills and required emotional labour. The skills created and sustained by introducing the CDSS included experiential, embodied and clinical expertise, and in turn this work offered an identity as ‘health workers’ and not as generic call centre operatives. This identity appears key to the recruitment and retention of these staff [62]. Deployment of the CDSS also disrupted some existing divisions of labour and hierarchies, for example, at 999 and SPA a new role – clinical supervisor – was introduced.

Although similar monitoring, appraisal and adaptation mechanisms keep the CDSS in place across the three settings, there were once again, differences in how these mechanisms were operationalised. For example the formal audit processes were undertaken differently in OOH, where the processes were more covert and focussed on identifying poor practice, compared with 999 and SPA where audit was seen more as a positive learning tool. Successful deployment of the CDSS entailed significant and long-term involvement from the developers including the need to adapt the system for each setting. One of the questions for further roll out of the CDSS will be how much this input can be reduced to implement the system at scale. It is worth noting that all three sites devoted considerable additional staff resources to support call-handlers, including clinical supervision (999 and SPA only) and audit and training staff.

## Conclusions

We have argued that implementing the CDSS and maintaining its everyday use was enabled by the collective action of a range of actors who established coherence and secured participation and engaged in on-going appraisal and adjustment to keep the technology in place. This effort was necessary to bring the CDSS into use and continues to be required to keep it working. As well as detailing how the same technology was implemented in three different settings this paper has demonstrated how NPT can be harnessed to provide a theoretical framework to structure cross-case analyses. Not everything that is technically possible will work in practice, but we can save time and money – and fulfil more hopes – if we can develop robust ways of understanding where effort invested is most likely to be successful. By using NPT we have been able to examine how sites differ in key respects – but also discern patterns and features which can help identify generative mechanisms that might, in turn, inform the implementation of these kinds of technologies in the future.

Our analysis has shown that the CDSS must be understood both as a computer technology and as a set of practices related to that technology, kept in place by a network of actors in particular contexts. The four domains of NPT - collective action, coherence, cognitive participation and reflexive monitoring play out differently in each setting even where the same technology is employed. The three settings are characterised by different ‘work’ and different workforce characteristics and while there might be a common core of training or skills, the content and format of this varies across the three settings. The ability and divisions of labour created and sustained by introducing the CDSS are not just those required to operate a computer system ‘by rote’ but are also about individual experiential, embodied expertise and team sharing of knowledge within a particular context or environment.

To our knowledge this is the first ethnographic study of a single CDSS used in three different emergency and urgent care settings. Our ethnography offers rich detail but like other case study research can be criticised as having limited generalizability. We have attempted to address this by deliberately employing a robust middle range theory (NPT) to provide an analytical framework that allows for comparison and explanation of how this technology was successfully brought into everyday, routine, use. Nonetheless we concede that the three settings studied may not be typical of similar services, and it remains for further research to test the reach of our interpretations. In addition we note that our research was located in particular moment in the development of the CDSS technology and of the life of each of the organisations studied. Some of what we observed was temporally contingent; for example, we suspect that the coherence and cognitive participation of future deployments of the CDSS will be strongly influenced by the sense-making and enrolment of the cases we studied as healthcare organisations learn from earlier adopters. A final limitation concerns the use of NPT. As noted earlier this theory is a relatively young middle range theory and our study is one of an emerging body of empirical applications of NPT. Other theoretical approaches might offer additional and no doubt different insights about the use of this particular CDSS. Our use of NPT means that we have paid less attention to the structures of networks keeping the technology in use (as Actor Network Theory might) or to organisational psychology (as the Theory of Planned Behaviour would). However we have demonstrated that this ICT has established some legitimacy amongst a range of users, who have been effectively enrolled in its deployment, who have collectively acted to bring it into use and who undertake the necessary reflexive tasks to keep it in play: in short we have explained how – unlike so many digital technologies in healthcare – the CDSS has been normalised.

Our research in these three different settings suggests that the CDSS has a strong chance of embedding in the long term and becoming routine in these services. However, we need to recognise that although single technologies can be implemented in different settings, this takes more effort than simply slotting a technology into place. Successful implementation has been achieved, and will only continue to be maintained, through the efforts of those involved in the specific settings and if the wider context continues to support the coherence, cognitive participation, and reflective monitoring processes that surround this collective action. Not least, technological interventions may require new resources to support their effective use, for example, requiring new roles, new organizational functions and considerable management time, all – perhaps – on an on-going basis.

## Abbreviations

: 999 Emergency /Ambulance service; CDSS: Computer Decision Support Systems; NPT: Normalisation Process Theory; OOH: Out of hours service; SPA: Single point of access to urgent care.

## Competing interests

The authors hold posts at University of Southampton, and the article processing charge will be paid by this higher education institution. CP, SH, JP, CM were grant holders on the National Institute for Health Research HS&DR grant which funded the research described here and JT and MC were employed as researchers on this project. The authors have no other financial or non-financial interests in relation to this manuscript.

## Authors’ contributions

CP, SH, JP, CM designed the study and secured funding. CP, SH, JP, JT, MC undertook data collection and analysis. CM advised on theoretical development. All authors contributed to the interpretations, contributed to the drafting and revising of this manuscript and have given final approval of the same.

## Pre-publication history

The pre-publication history for this paper can be accessed here:

http://www.biomedcentral.com/1472-6963/13/111/prepub
